# The Outcomes of the Patients Undergoing Harmonic Scalpel Laparoscopic Cholecystectomy

**DOI:** 10.7759/cureus.15622

**Published:** 2021-06-13

**Authors:** Amudhan Kannan, Anjli Tara, Huma Quadir, Knkush Hakobyan, Mrunanjali Gaddam, Ugochi Ojinnaka, Zubayer Ahmed, Jerry Lorren Dominic, Ketan Kantamaneni, Terry R Went, Jihan A Mostafa

**Affiliations:** 1 General Surgery, California Institute of Behavioral Neurosciences & Psychology, Fairfield, USA; 2 General Surgery, Stony Brook Medicine, Southampton Hospital, Southampton, USA; 3 Internal Medicine/Family Medicine, California Institute of Behavioral Neurosciences & Psychology, Fairfield, USA; 4 Neurology, California Institute of Behavioral Neurosciences & Psychology, Fairfield, USA; 5 Diagnostic Radiology, California Institute of Behavioral Neurosciences & Psychology, Fairfield, USA; 6 Internal Medicine, California Institute of Behavioral Neurosciences & Psychology, Fairfield, USA; 7 Family Medicine, California Institute of Behavioral Neurosciences & Psychology, Fairfield, USA; 8 General Surgery/Orthopedic Surgery, Cornerstone Regional Hospital, South Texas Health System, Edinburg, USA; 9 Vascular Surgery, California Institute of Behavioral Neurosciences & Psychology, Fairfield, USA; 10 Psychiatry, California Institute of Behavioral Neurosciences & Psychology, Fairfield, USA

**Keywords:** laparoscopic cholecystectomy, conventional laparoscopic cholecystectomy, harmonic scalpel, clip-less cholecystectomy, patient outcomes

## Abstract

Laparoscopic cholecystectomy has replaced conventional open cholecystectomy and has become the gold standard surgery for gall bladder pathologies. The harmonic scalpel is one of the instruments used to dissect and coagulate. Most surgeons accept the usage of the harmonic scalpel in laparoscopic cholecystectomy. The other standard method is electrocoagulation by electrocautery. The harmonic scalpel cholecystectomy has several advantages over other methods of laparoscopic cholecystectomy. Electrocoagulation by electrocautery produces smoke which can result in damage to lateral tissues, including the gall bladder. The clips are used along with electrocoagulation to seal cystic duct and cystic artery before dissection. There are various studies about bile leakage in the case of clip application. The harmonic scalpel uses ultrasonic energy to achieve hemostasis without bleeding, dissection, and gallbladder removal from the liver bed during laparoscopic surgery by causing coagulation of proteins. The patient outcome variables such as postoperative pain, duration of hospital stay, postoperative nausea and vomiting, surgical site infections, and other complications have not been compared in review articles. In this review, we collected the information from previously published studies and reviewed the outcomes of patients undergoing harmonic scalpel cholecystectomy. Harmonic scalpel cholecystectomy reduces the duration of hospital stay, duration of operation, intraoperative and postoperative complications, and postoperative pain. Thus the harmonic scalpel can be used instead of other instruments as it has better patient outcomes.

## Introduction and background

The first person to perform laparoscopic cholecystectomy was Professor Muhe of Boblingen, Germany. He performed the operation on September 12, 1985. Since then, laparoscopic cholecystectomy has undergone various moldings, changes, and add-ons. This includes the use of multiple techniques for coagulation during dissection. Now, laparoscopic cholecystectomy has replaced conventional open cholecystectomy and has become the gold standard. Indications for cholecystectomy include cholecystitis (acute and chronic), biliary dyskinesia, and gall stones diseases. The coagulation during dissection can be achieved by conventional electrocoagulation or the harmonic scalpel, which uses ultrasonic energy. Most Surgeons accept harmonic scalpel use in laparoscopic cholecystectomy [1].

Electrocoagulation by electrocautery produces smoke and can result in damage to lateral tissues [1]. Titanium clips are used along with electrocoagulation to seal cystic duct and cystic artery before dissection. There are various studies about bile leakage in the case of titanium clip application. The harmonic scalpel uses ultrasonic energy. Ultrasonic energy coagulates proteins to achieve hemostasis without bleeding, dissection, and gallbladder removal from the liver bed [2]. Studies have shown that the harmonic scalpel is safe, requires shorter operative time, involves less heat and smoke production, less incidence of gall bladder perforation, and less conversion rate to open cholecystectomy [3]. Reviews have not studied the patient outcomes in harmonic scalpel cholecystectomy (HSC). 

Various studies have compared harmonic scalpel and electrocoagulation on multiple variables, such as intraoperative bleeding, postoperative bile leakage, duration of operation, etc. The patient outcome variables, which are essential in terms of patient wellbeing, have not been compared in literature reviews. Abounozha et al. have already done studies that showed that the risk of bile leakage and intra-operative bleeding in HSC is comparatively less compared to electrocoagulation [4,5]. Ng et al. have done a literature review about the titanium clip, which is usually used in electrocoagulation, acting as a nidus for stone formation for choledocholithiasis [6]. Schreuder et al. and Angelescu et al. have also reported similar cases of clips acting as a nidus for stone formation [7,8]. The studies by Eltiras et al. and Sharma et al. had shown that the duration of the operation is less in HSC [9,10].

The patient outcome variables such as postoperative pain, duration of hospital stay, postoperative nausea and vomiting, surgical site infections, and other complications have not been compared in review articles. The outcome variables are essential in terms of patient satisfaction and wellbeing after the operation.

In this review, we collected the information from previously published articles and study the outcomes of patients undergoing HSC such as postoperative pain (using the visual analog scale {VAS} score postoperatively/use of analgesics), postoperative nausea/vomiting, surgical site infections, duration of hospital stay, other complications in patients who underwent HSC.

## Review

The harmonic scalpel has many advantages over other instruments used for laparoscopic cholecystectomy. The outcomes such as postoperative pain, duration of hospital stay, and time to resume a regular diet were studied and compared.

Postoperative pain depends on the duration of operation and bile leak

**Figure 1:**
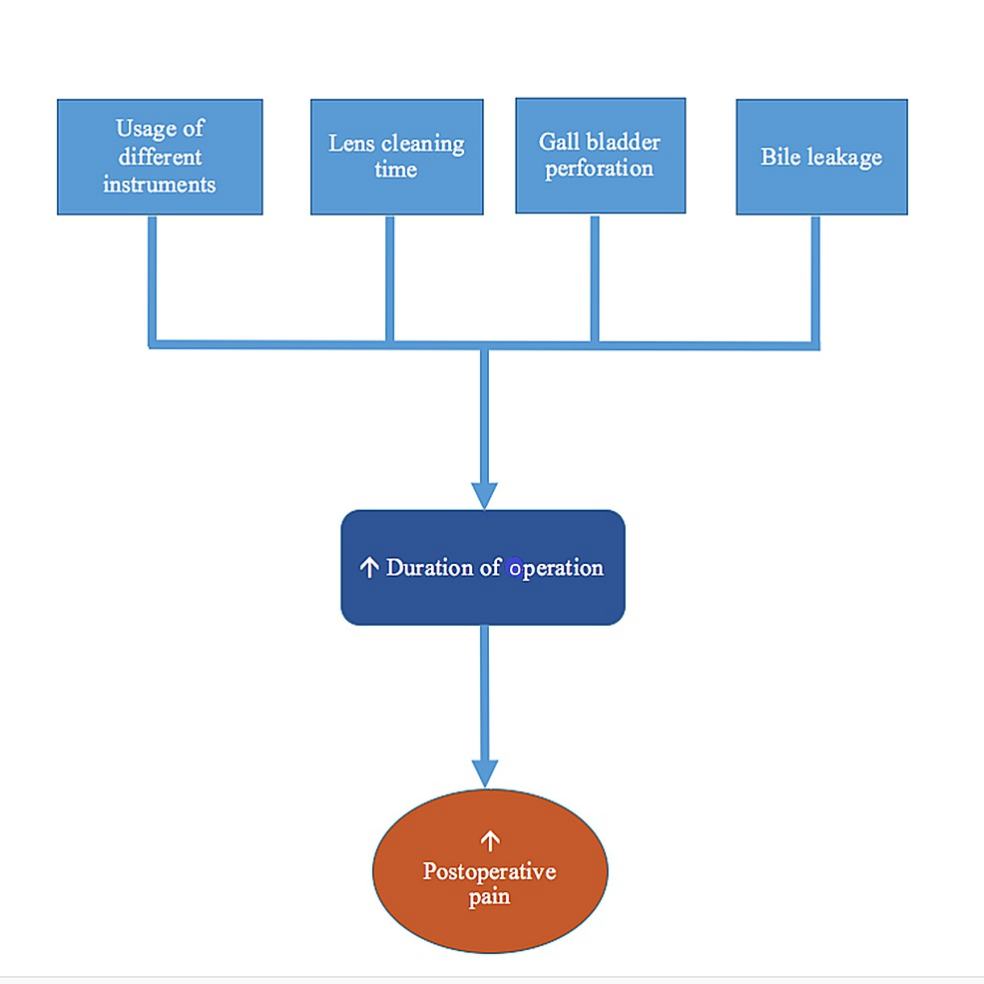
Factors contributing to postoperative pain

There are various mechanisms for how pain occurs after laparoscopic cholecystectomy. One of the mechanisms is the leakage of bile into the peritoneum leading to irritation of the peritoneum and thus causing pain [1]. Bile can leak in case of incomplete coagulation of ducts or case of gall bladder perforation. Pain also occurs because of pneumoperitoneum. The mechanisms of postoperative pain in laparoscopic cholecystectomy are presented in Figure 1.

HSC and Duration of Operation

In HSC, only one instrument is used: the harmonic scalpel compared to other coagulation methods. Many instruments have to be taken and disposed of in other methods, which contributes to delay, leading to increased operation duration [11]. When used, the Harmonic scalpel replaces four common instruments used in the laparoscopic cholecystectomy viz. scissors, dissector, clip applier, and electrosurgical hook.

Bessa et al. conducted a prospective study of 120 patients with gall bladder disease undergoing laparoscopic cholecystectomy [12]. These patients were assigned either to the harmonic scalpel group (HS group = 60) or the clip and cautery group (C&C = 60). The duration of operation was significantly shorter in the harmonic scalpel group. The duration of operation in the harmonic scalpel group was again significantly shorter in the absence of gallbladder perforation. In the presence of gall bladder perforation, the duration of operation did not show a significant difference when the harmonic scalpel was used. But the incidence of gallbladder perforation was less in the harmonic scalpel group [12].

Mahabaleshwar et al. conducted a randomized controlled trial of 60 patients with gall bladder disease undergoing laparoscopic cholecystectomy [13]. These patients were randomly assigned into either the harmonic scalpel group (HS group = 30) or the electrocautery group (electrocautery group = 30). The lens cleaning time was significantly longer in the electrocautery group. The duration of surgery was also significantly longer in the electrocautery group [13].

Electrocautery produces smoke which can obscure the camera and thus affecting visibility. The produced smoke has to be taken out by opening trocar, which will lead to loss of the pneumoperitoneum multiple times and contributes to the delay [13]. The camera has to be taken out to clear the smoke from the field, contributing to a delay in operation. The harmonic scalpel does not produce smoke, and thus there will not be a situation to clear the camera field. Thus, the harmonic allows the surgeon to operate in a clear operative field, reducing the duration of the operation.

HSC and Bile Leakage

The clips are used to seal the cystic duct after gall bladder removal when the electrocoagulation method is used for surgery. Many studies and reports have shown that the clips do not lead to complete closure of the cystic duct and can slide down, leading to leakage of bile both intra-operatively and post-operatively. Many studies have shown that the clip can act as a nidus for subsequent stone formation [6,8].

The harmonic scalpel is safe and effective in the closure of the cystic duct. HSC does not need the application of the clip. Therefore, it is also called a "clipless cholecystectomy." Abounozha et al. had done a review to determine whether bile leak was higher in patients undergoing harmonic scalpel cholecystectomy. The authors studied six articles that met their inclusion criteria. The authors did not find a significantly higher bile leak rate in all their included studies and concluded that the clipless laparoscopic cholecystectomy was a safe and practical technique in preventing bile leakage [4].

HSC and Gall Bladder Perforation

Electrocoagulation and dissection cause damage to lateral tissue. The risk of perforation of the gall bladder is more as lateral damage to the gallbladder can occur while dissecting its bed. If gall bladder perforation occurs, then the bile has to be evacuated from the peritoneum, leading to an increased surgery duration. Moreover, the minor bile, which cannot be evacuated by any means, can irritate the peritoneum, causing pain. Patients with complications such as adhesions, fibrotic gall bladder, cholecystitis have an increased risk of perforation of the gall bladder when electrocoagulation is used for dissection [14]. The harmonic scalpel effectively dissects adhesions, fibrotic gall bladder, and inflamed gall bladder.

Janssen et al. conducted a prospective study of a total of 200 patients undergoing laparoscopic cholecystectomy [14]. With the exclusion of one patient, the others were assigned randomly to either the harmonic scalpel group (n=96) or the electrocautery group (n=103). In this study, the harmonic scalpel usage was associated with less incidence of gall bladder perforation. This study also found that the surgeon’s experience did not influence the perforation rate within the harmonic scalpel group. But in the electrocautery group, the incidence of gall bladder perforation varied with the surgeon’s experience, and it was significantly higher. The authors concluded that harmonic scalpel cholecystectomy is the technique of choice for gallbladder dissection in patients with complications [14]. Downes et al. studied a total of 28 patients who underwent single-incision laparoscopic cholecystectomy with the usage of a harmonic scalpel for dissection and coagulation. Only two cases of gallbladder perforation occurred [15].

As HSC is associated with less risk for gall bladder perforation, less risk of bile leakage, and shorter duration of surgery, postoperative pain is less when the harmonic scalpel is used. Postoperative pain contributes to patient’s morbidity and duration of hospital stay. Patients might have to be put on various analgesics if the pain is significant, and this will cause inconvenience. As HSC is associated with less postoperative pain, Patients can be well managed with low dose analgesics, and thus the duration of hospital stay and morbidity will be less.

HSC and duration of hospital stay

**Figure 2:**
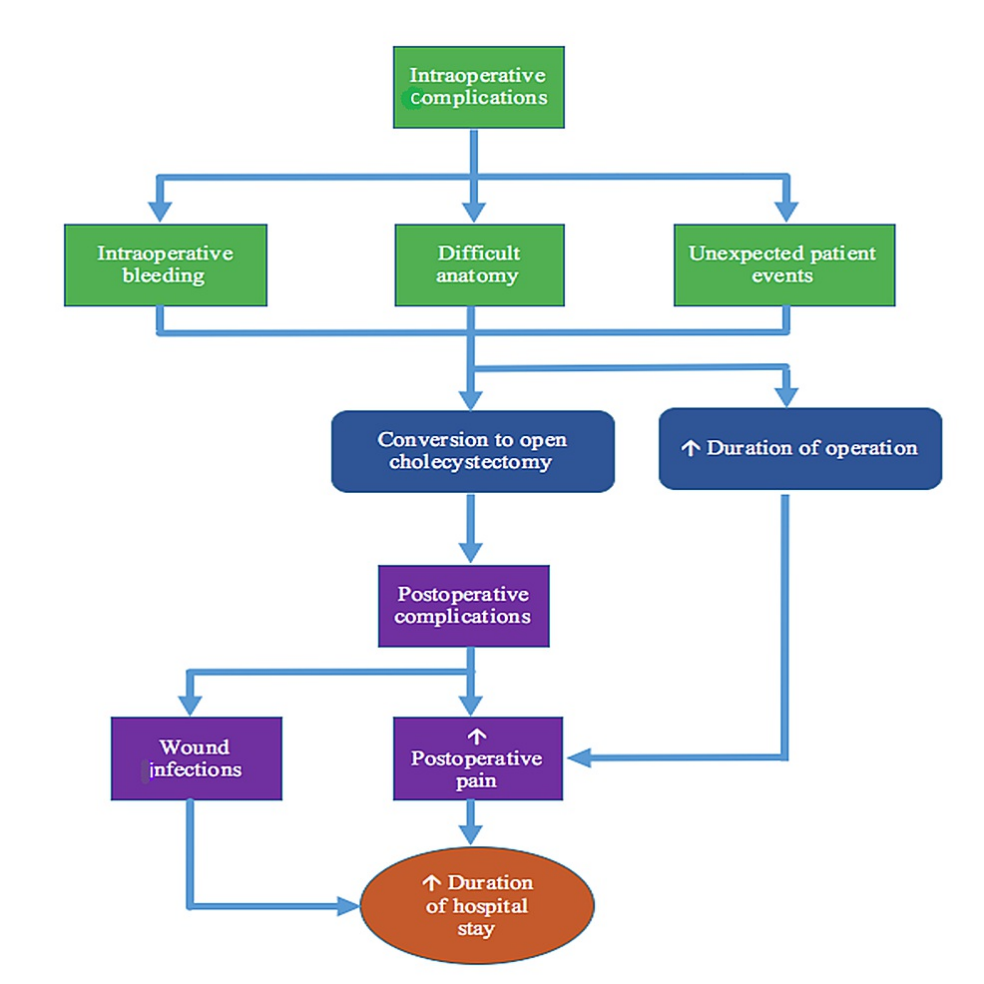
Factors contributing to increased duration of hospital stay

The duration of hospital stay in patients undergoing laparoscopic cholecystectomy depends on many things. It depends on: the postoperative pain, complications such as wound infections, conversion to open cholecystectomy, bile leakage-related complications, other infections, and other complications of the surgery. As already seen, the risk of bile leakage and gall bladder perforation is less when the harmonic scalpel is used. The mechanisms contributing to increased duration of hospital stay in laparoscopic cholecystectomy are presented in Figure 2.

HSC and Conversion Rates

Conversion to open cholecystectomy is done when surgical complications occur intraoperatively. The most common reason for conversion to open cholecystectomy is intraoperative bleeding. The harmonic scalpel is safe as it provides complete hemostasis and is a safe alternative to the standard clipping of the cystic artery.

Catena et al. had done a randomized controlled trial [16]. A total of 42 patients were assigned randomly to either the harmonic scalpel group (H = 21) or the monopolar diathermy group (MD = 21). The intraoperative blood loss was significantly less in the harmonic scalpel group. The conversion rate was also significantly less in the harmonic scalpel group [16]. In the same study, the reduction in conversion and blood loss was evident in technically demanding cases with anatomical difficulty when the harmonic scalpel was used [16].

Abounozha et al. had done a study to determine whether HSC is associated with less intraoperative bleeding. Five studies that met their inclusion criteria were included. The authors concluded that the harmonic scalpel effectively controls bleeding and can be used in patients with a high risk of intraoperative bleeding [5]. When the harmonic scalpel is used, the conversion rate is less when compared to other techniques [2,16].

HSC and Surgical Site Infections

The studies included in this review have shown that reduction in surgical site infection was comparable when the harmonic scalpel is used. Many studies also reported that surgical site infection incidence had no change when the harmonic scalpel was used [3,12,13]. As conversion rates are lower when the harmonic scalpel is used, the risk of surgical site infection and skin wound infections will be lower. On the whole, harmonic scalpel usage is associated with significantly less complications when compared to other methods of coagulation and dissection.

Almost all patients want to resume their normal state and go home to resume their normal life in a healthy state as soon as possible after the operation. The complications cause a delay in the discharge and thus leading to a prolonged hospital stay. Patients also develop hospital stay-related complications such as iatrogenic infections, which adds to morbidity. Conversion to open cholecystectomy will almost always lead to a prolonged hospital stay as we need to need to monitor patients for postoperative complications of open surgeries. Wound infections commonly occur in open surgeries. By reducing the conversion rate, HSC reduces the duration of hospital stay of the patients [3,9,17].

Resumption to the normal diet

The patients undergoing laparoscopic cholecystectomy often have postoperative nausea and vomiting. This is due to irritation of the peritoneum and damage caused by the smoke when electrocautery is used. Nausea and vomiting can also be due to recovery from anesthesia. But if bile-induced peritoneal irritation occurs, nausea and vomiting are prolonged, leading to a delay in resumption to a regular diet. This poses an important issue for patients as they cannot tolerate oral feeds, and intravenous fluids must be administered.

Kandil et al. had done a prospective randomized study of 140 patients undergoing laparoscopic cholecystectomy who were randomly assigned to either the diathermy group (n=70) or the harmonic scalpel group (n=70). The incidence of nausea and vomiting was higher in the diathermy group. But it did not show a significant difference [3].

El Nakeeb et al. had done a prospective study of a total of 120 patients who underwent laparoscopic cholecystectomy. These patients were randomly assigned to either the harmonic scalpel group (n=60) or the traditional method (n=60). The results showed that the harmonic scalpel reduces the time taken for resumption to a regular diet [18]. The studies included in this review had shown a comparable reduction in nausea and vomiting when the harmonic scalpel was used [3,9].

HSC in patients with liver cirrhosis

Patients with liver cirrhosis have a high risk for gallstone formation and cholecystitis more than non-cirrhotic patients [2,19]. These patients have a higher incidence of gallstone disease. Patients with liver cirrhosis have another problem which is bleeding diastasis. More care should be taken not to injure the cystic artery or blood vessel in patients with liver cirrhosis. If not, it will lead to intra-operative bleeding, thereby leading to conversion.

Bessa et al. had done a prospective randomized study of 40 cirrhotic Child-Pugh’s classes A and B patients with gallstone disease. These patients were randomly assigned to either the harmonic scalpel group (n = 20) or the conventional diathermy group (n = 20). The intraoperative blood loss and incidence of gall bladder perforation were significantly less in the harmonic scalpel group. All procedures were completed by the laparoscopic method in the harmonic scalpel group. There was a failure to control bleeding in three patients in the conventional diathermy group, which needed conversion [20].

Open cholecystectomy in patients with liver cirrhosis has high morbidity and mortality [3]. Duration of operation also contributes to morbidity. The risk of bleeding increases with an increase in the duration of the operation. The harmonic scalpel usage in patients with liver cirrhosis provides a shorter operative time and less intraoperative bleeding when compared to other techniques [9,18,20].

Disadvantages of HSC

Like any other method, the harmonic scalpel cholecystectomy is not there without any disadvantages. The disadvantages include the cost of the instrument: the harmonic scalpel, and the difference in patient outcomes when the harmonic scalpel is used by surgeons-in-training and expert surgeons.

Cost of the Instrument Harmonic Scalpel

The disadvantage of HSC is none other than the cost of the instrument harmonic scalpel. The cost of the harmonic scalpel becomes essential when the hospital is provided with reusable instruments.

Some studies have shown that the combined cost of using multiple disposable instruments such as dissector, scissors, clipper, electrocautery hook, and grasper is more than using the harmonic scalpel alone. In the study by Hüscher et al., cost analysis of disposable devices in standard laparoscopic cholecystectomy and HSC did not significantly differ [21]. More studies are needed to compare the cost of HSC and other methods of laparoscopic cholecystectomy.

Surgeons-in-Training Versus Surgeon Expert on Patient’s Outcome

The study by Hüscher et al. had shown that the usage of the harmonic scalpel for cholecystectomy by surgeons-in-training showed a reduction in the duration of operation and no significant reduction when used by the expert subgroup [21]. Further studies to determine the effectiveness of harmonic scalpel usage by surgeons-in-training in reducing the operative time have to be done.

Limitations of this review

This review has included only a limited number of studies due to the non-availability of full articles. Many studies used in the review had small sample sizes. Most of them were randomized clinical trials, and so the sample size was limited. This review has included studies only from the year 2000 to 2021.

## Conclusions

The primary objective of this review was to study the outcomes of patients who underwent laparoscopic cholecystectomy using the harmonic scalpel. Harmonic scalpel cholecystectomy reduces the duration of hospital stay, duration of operation, intraoperative and postoperative complications, and postoperative pain. End of the day, patient satisfaction and wellbeing are crucial for any surgery. There are no reviews about the outcomes of patients who underwent harmonic scalpel cholecystectomy to the best of our knowledge. Thus instead of other instruments, the harmonic scalpel can be used as it has better patient outcomes. The authors of this review recommend further clinical trials comparing the surgeon-in-training and expert’s performance in patient outcomes when harmonic scalpel and other instruments are used.
